# Computational Model of Recurrent Subthalamo-Pallidal Circuit for Generation of Parkinsonian Oscillations

**DOI:** 10.3389/fnana.2017.00021

**Published:** 2017-03-21

**Authors:** Osamu Shouno, Yoshihisa Tachibana, Atsushi Nambu, Kenji Doya

**Affiliations:** ^1^Okinawa Institute of Science and Technology Graduate UniversityOkinawa, Japan; ^2^Honda Research Institute Japan Co., Ltd.Saitama, Japan; ^3^Division of System Neurophysiology, Department of Physiological Sciences, National Institute for Physiological Sciences, Graduate University for Advanced StudiesAichi, Japan

**Keywords:** basal ganglia, parkinsonian oscillation, subthalamic nucleus, globus pallidus, beta oscillation, rebound excitaton, short-term plasticity

## Abstract

Parkinson's disease is a movement disorder caused by dopamine depletion in the basal ganglia. Abnormally synchronized neuronal oscillations between 8 and 15 Hz in the basal ganglia are implicated in motor symptoms of Parkinson's disease. However, how these abnormal oscillations are generated and maintained in the dopamine-depleted state is unknown. Based on neural recordings in a primate model of Parkinson's disease and other experimental and computational evidence, we hypothesized that the recurrent circuit between the subthalamic nucleus (STN) and the external segment of the globus pallidus (GPe) generates and maintains parkinsonian oscillations, and that the cortical excitatory input to the STN amplifies them. To investigate this hypothesis through computer simulations, we developed a spiking neuron model of the STN-GPe circuit by incorporating electrophysiological properties of neurons and synapses. A systematic parameter search by computer simulation identified regions in the space of the intrinsic excitability of GPe neurons and synaptic strength from the GPe to the STN that reproduce normal and parkinsonian states. In the parkinsonian state, reduced firing of GPe neurons and increased GPe-STN inhibition trigger burst activities of STN neurons with strong post-inhibitory rebound excitation, which is usually subject to short-term depression. STN neuronal bursts are shaped into the 8–15 Hz, synchronous oscillations via recurrent interactions of STN and GPe neurons. Furthermore, we show that cortical excitatory input to the STN can amplify or suppress pathological STN oscillations depending on their phase and strength, predicting conditions of cortical inputs to the STN for suppressing oscillations.

## Introduction

Parkinson's disease (PD) is characterized by progressive movement disorders caused by dopamine depletion in the basal ganglia (BG) resulting from degeneration of midbrain dopaminergic neurons. Abnormally synchronized oscillations in local field potentials (LFPs) between 8 and 30 Hz, which are called “the BG beta frequency band” (Hammond et al., 2007), are observed in the subthalamic nucleus (STN) and the internal/external segments of the globus pallidus (GPi/GPe) of PD patients (Miller and DeLong, 1987; Levy et al., 2001; Kühn et al., 2005). These abnormal oscillations are implicated in PD symptoms, such as akinesia, bradykinesia, and rigidity (Boraud et al., 2002; Brown, 2003; Gatev et al., 2006; Rivlin-Etzion et al., 2006; Hammond et al., 2007; but see Leblois et al., 2006b). We constructed a spiking neuron model of the recurrent STN-GPe circuit and investigated mechanisms for generation of parkinsonian beta band oscillations by exploring physiological parameters.

BG beta oscillations in LFPs are divided into upper-beta (>20 Hz) and lower-beta (<20 Hz) oscillations, and only the lower-beta oscillations are sensitive to dopaminergic therapy (Priori et al., 2004). Single-unit recordings from the STN in human PD patients show abnormal neuronal burst oscillations in the 10–25 Hz frequency band, which could be the source of pathological LFP oscillations (Levy et al., 2001, 2002). Neuronal recordings in nonhuman primate PD models show exaggerated oscillatory burst activities in STN, GPe, and GPi that are concentrated in narrow frequency bands of 4–8 Hz and 8–15 Hz (Raz et al., 2000; Heimer et al., 2006; Wichmann and Soares, 2006; Tachibana et al., 2011), the latter of which overlaps with the frequency band recorded from human PD patients. These 8–15 Hz neuronal burst oscillations are reduced by both dopaminergic therapy and deep brain stimulation, which can alleviate PD motor symptoms (Heimer et al., 2006; Rosin et al., 2011; Tachibana et al., 2011). Recently, neuronal recordings from primate PD models under a variety of pharmacological manipulations demonstrated that STN 8–15 Hz neuronal oscillations are the origin of GPe/GPi oscillations and that they are dependent on GABAergic inhibition from GPe and glutamatergic inputs from the cortex/thalamus (Tachibana et al., 2011).

Computational modeling studies have tackled mechanisms of pathological BG oscillations (Gillies et al., 2002; Terman et al., 2002; Rubin and Terman, 2004; Leblois et al., 2006a; Hahn and McIntyre, 2010; Holgado et al., 2010; Kumar et al., 2011). However, their simulated results did not agree well with experimentally-observed neuronal properties of the BG 8–15 Hz burst oscillations (Wichmann and Soares, 2006; Tachibana et al., 2011). Furthermore, the proposed role of strengthened striatal inhibition for the induction of the BG oscillations (Terman et al., 2002; Kumar et al., 2011) is not consistent with the experimental evidence that the blockade of GABAergic synaptic transmissions of GPe neurons from the striatum and other GPe neurons did not affect the 8–15 Hz oscillations (Tachibana et al., 2011). Therefore, the manner in which the pathological 8–15 Hz burst oscillations are generated remains unclear. Based on experimentally-observed neuronal data (Tachibana et al., 2011), we investigated mechanisms for generation and maintenance of pathological 8–15 Hz burst oscillations in recurrent STN-GPe circuits in a dopamine-depleted state. Using computer simulations of a spiking neuron model of the recurrent STN-GPe circuit, we explored the required neuronal, synaptic, and circuit mechanisms for normal firing and pathological burst oscillation. We then investigated how cortical excitatory input to the STN affects BG oscillations generated in the STN-GPe circuit and showed that for two parameter regions, the model reproduces BG neuronal activity in terms of mean firing rates, oscillation frequency, and burst discharges, corresponding to normal and parkinsonian states. The same mechanism for generation of BG pathological oscillations also predicts suppressive effects of non-oscillatory cortical inputs to the BG oscillations that explains suppression of pathological BG oscillations by movements (Amirnovin et al., 2004; Brown and Williams, 2005). We further demonstrated that oscillatory inputs from the cortex to the STN can amplify or diminish pathological BG oscillations in a phase-dependent manner, and can predict necessary conditions for their suppression.

## Materials and methods

### The model

Experimental evidence suggests that reciprocal STN-GPe interconnections and glutamatergic inputs to the STN in dopamine-depleted states are both important for generation and amplification of the parkinsonian, oscillatory burst activity of STN neurons that subsequently propagates to the GPi (Tachibana et al., 2011). Post-inhibitory rebound excitation of STN neurons is a candidate driving force of the oscillatory burst activity (Bevan et al., 2002), which could account for the importance of GPe inhibition to the STN for generation of the BG parkinsonian oscillatory burst activity of STN neurons (Tachibana et al., 2011). Short-term plasticity at excitatory synapses of GPe neurons and at GABAergic synapses of STN neurons are implicated in modulating temporal patterns of GPe and STN neurons in the normal state, and as well as in the parkinsonian state (Hanson and Jaeger, 2002; Atherton et al., 2013). Dopamine depletion in the BG is the key phenomenon for the induction of parkinsonian symptoms and BG pathological oscillations (Heimer et al., 2006; Wichmann and Soares, 2006; Tachibana et al., 2011). We considered the following neural phenomena that are related to BG dopamine depletion as candidate causes of the BG pathological oscillations: (1) reduction of GPe autonomous activity (Chan et al., 2011), (2) strengthening of GPe inhibition to the STN due to release from D2-receptor-mediated presynaptic suppression of transmitter release (Shen and Johnson, 2000, 2005), and by an increase in the number of synaptic connections per GPe-STN axon terminal (Fan et al., 2012), (3) increased STN-GPe excitation to compensate for the reduction of GPe autonomous activity (Chan et al., 2011), and (4) loss of active decorrelation ability of GPe neurons (Edgerton and Jaeger, 2011). Although elevated striatal inhibition of GPe neurons by dopamine depletion (Hernandez-Lopez et al., 2000; Liang et al., 2008) is often linked to pathological BG oscillations (Terman et al., 2002; Kumar et al., 2011), we excluded it from candidate neural phenomena related to generation of BG oscillations because experimental results showed that blockade of ionotropic GABAergic transmission either did not affect or even exaggerated GPe 8–15 Hz oscillations (Tachibana et al., 2011). Based on this evidence, we focused on a model of closed STN-GPe circuits incorporating the STN post-inhibitory rebound mechanism and dynamic synapses as mechanisms for generation of parkinsonian BG oscillations in the dopamine-depleted state. First, we explored required conditions of GPe autonomous activity and of connection strengths between the STN and GPe for generation of BG oscillations in the closed STN-GPe circuits. Later, we investigated roles of cortical excitatory inputs in the STN for BG oscillations that were generated in closed STN-GPe circuits.

### Model architecture

**Figure 1:**
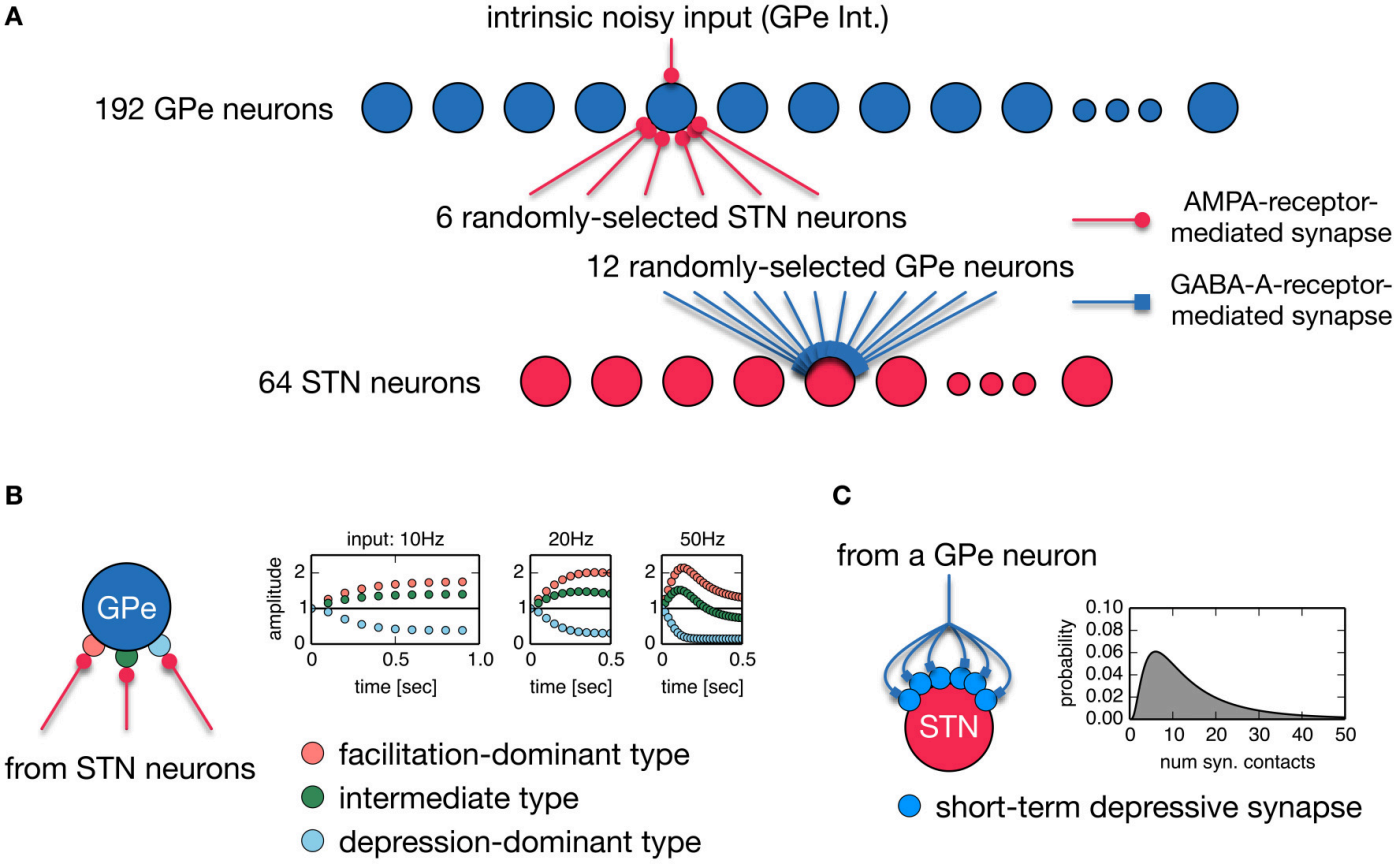
**(A)** A random, sparsely-connected architecture of the recurrent circuit model between the STN and GPe. **(B)** Properties of three different types of short-term plasticity at STN-GPe synapses. A type of short-term plasticity is randomly chosen for a STN-GPe synapse with equal probability from these three types regardless of a post-synaptic neuron. **(C)** A single GPe axon gives multiple synaptic contacts on a STN neuron. Transmission probability of a unitary GPe projection is dynamically modulated by short-term depression. The number of synaptic contacts that a single GPe axon established on an STN neuron obeys the log-normal distribution.

Anatomically, the STN and GPe are reciprocally connected. STN neurons are a major source of excitatory inputs to the GPe, and GPe neurons are a major source of inhibitory inputs to the STN. These connections are very sparse and their average connectivity is low (Kita and Kitai, 1994; Bevan et al., 1998; Sato et al., 2000; Baufreton et al., 2009). Based on these findings, we developed a random, sparsely-connected architecture of the recurrent circuit model between the STN an GPe. The architecture of our network model is shown in Figure 1A. The model consisted of 64 STN neurons and 192 GPe neurons unless otherwise noted. Each STN neuron received GABAergic projections from 12 randomly-selected GPe neurons. GPe-STN connectivity is of the same order of magnitude as experimental results (Baufreton et al., 2009). Each GPe neuron received glutamatergic projections from 6 randomly selected STN neurons. We did not include GABAergic connections between GPe neurons in our model because they were controversial: Anatomical studies showed inhibitory axonal collaterals of GPe neurons (Bevan et al., 1998; Sato et al., 2000), while functional interactions between GPe neurons were very weak or nonexistent. Each glutamatergic projection onto a GPe neuron gave a single synaptic contact, and exhibited short-term plasticity (Hanson and Jaeger, 2002; Figure 1B). Each GABAergic projection from the GPe onto an STN neuron gave *N* synaptic contacts that were randomly drawn from a log-normal distribution of mean = 15.50 and standard deviation = 14.53 (Figure 1C), which fit experimental data well (Baufreton et al., 2009). Each GPe neuron had intrinsic noisy conductance (GPe Int.), as will hereinafter be described in detail. A population of cortical excitatory inputs to the STN was modeled as a stochastic spike train that obeyed a Poisson process, the mean rate of which was constant at 100 Hz, unless otherwise noted. GPe-STN GABAergic synapses exhibited short-term depression (Atherton et al., 2013). Axonal conduction delays of STN-GPe, and GPe-STN were 5 ms (Fujimoto and Kita, 1993; Kita et al., 2005).

### Neuron and synapse models

An STN neuron was simulated by using a single-compartment, conductance-based model (Otsuka et al., 2004) that contains several sets of currents:

$$\begin{array}{l} \begin{array}{l} C_{m}\frac{dV}{dt}=-I_{Na}-I_{K}-I_{A}-I_{LCa}-I_{T}-I_{Ca-K}-I_{leak}\\ \;-\;\frac{g_{AMPA}}{A_{m}}(V-V_{exc})-\frac{g_{GABA}}{A_{m}}(V-V_{inh}), \end{array}\\ I_{Na}=g_{Na}m^{3}h(V-V_{Na}),\\ I_{K}=g_{K}n^{4}(V-V_{K}),\\ I_{A}=g_{A}a^{2}b(V-V_{K}),\\ I_{LCa}=g_{LCa}c^{2}d_{1}d_{2}(V-V_{Ca}),\\ I_{T}=g_{T}p^{2}q(V-V_{Ca}),\\ I_{leak}=g_{leak}(V-V_{leak}) \end{array}$$

where *C*_*m*_ is membrane capacitance and takes 1 μF/cm^2^. *V* is the membrane potential. *I*_*Na*_ is a voltage-dependent sodium current. *I*_*K*_ is a voltage-dependent, delayed rectifier potassium current. *I*_*A*_ is an A-type potassium current. *I*_*LCa*_ is an L-like, long-lasting calcium current. *I*_*T*_ is a low threshold T-type calcium current. *I*_*Ca*−*k*_ is a calcium-activated potassium current. *a, b, c, d1, d2, h, m, n, p, q*, and *r* are activation or inactivation variables of these ionic currents. *V*_*Na*_, *V*_*K*_, and *V*_*Ca*_ are the equilibrium potentials of sodium, potassium, and calcium, respectively. *V*_*leak*_ is the resting potential of the leak current. *V*_*exc*_ and *V*_*inh*_ are excitatory and inhibitory synaptic reversal potentials. *g*_*Na*_, *g*_*K*_, *g*_*A*_, *g*_*LCa*_, *g*_*T*_, and *g*_*leak*_ are the maximal conductances of *I*_*Na*_, *I*_*K*_, *I*_*A*_, *I*_*LCa*_, *I*_*T*_ and *I*_*leak*_, respectively. Parameter values of the STN model cell were identical to the original ones except for excitatory and inhibitory synaptic reversal potentials (*V*_*exc*_ = 0.0 mV and *V*_*inh*_ = −84.0 mV), and the surface area of a cell, *A*_*m*_. Although the original model is for a unit-surface area of membrane, we set *A*_*m*_ = 0.2 μm^2^ so as to match the experimentally observed relationship between firing rate and applied current intensity (Otsuka et al., 2004). Excitatory synaptic conductance mediated by AMPA-type glutamatergic receptors, *g*_*AMPA*_, was modeled as the alpha function:

$$g_{AMPA}\left(t\right)={\bar{g}}_{AMPA-Ctx-STN}\frac{t}{\tau_{AMPA}}exp\left(-\frac{t}{\tau_{AMPA}}\right).$$

We chose a synaptic time constant and the maximal synaptic conductance of an excitatory synapse as τ_*AMPA*_ = 1.0 ms, ḡ_*AMPA*−*Ctx*−*STN*_ = 1.0 nS. Although a physiological study shows that an STN neuron receives a substantial number of excitatory inputs via NMDA-type glutamatergic receptors in addition to AMPA-type-receptor-mediated inputs (Nambu et al., 2000), we did not simulate NMDA-type conductance in this study because preliminary simulation results did not show any significant differences with or without it. The GABAergic synaptic conductance, *g*_*GABA*_, was modeled as the beta function:

$$g_{GABA}\left(t\right)={\bar{g}}_{GABA-GPe-STN}\left(exp\left(-\frac{t}{\tau_{decay}}\right)-exp\left(-\frac{t}{\tau_{rise}}\right)\right).$$

We chose τ_*decay*_ = 7.7 ms and τ_*rise*_ = 0.380 ms for GABAergic synapses to fit experimental data (Baufreton et al., 2009). A GPe neuron was implemented as a single-compartment conductance-based leaky integrate-and-fire model:

$$C_{m}\frac{dV}{dt}=-g_{leak}\left(V-V_{leak}\right)-g_{AMPA}\left(V-V_{exc}\right).$$

Parameter values of GPe model neurons were as follows: *C*_*m*_ = 0.25 pF, *g*_*leak*_ = 16.66 nS, *V*_*leak*_ = −70.0 mV, *V*_*exc*_ = 0.0 mV. The absolute refractory period was set to 2.0 ms. Threshold and reset potentials were −55.0 and −60.0 mV, respectively. Synaptic conductances of GPe neurons were modeled as the beta function, after Baufreton et al. (2009):

$$\begin{array}{l} g_{AMPA}\left(t\right)=\left({\bar{g}}_{AMPA-STN-GPe}+\;{\bar{g}}_{AMPA-GPe-Int}\right)\\ \text{​​​​​​​​​ ​}\left(exp\left(-\frac{t}{\tau_{decay}}\right)-exp\left(-\frac{t}{\tau_{rise}}\right)\right) \end{array}$$

We chose τ_*decay*_ = 12.4 ms and τ_*rise*_ = 5.0 ms (Hanson and Jaeger, 2002). ḡ_*AMPA*−*STN*−*GPe*_ and ḡ_*AMPA*−*GPe*−*Int*_ represent the maximal synaptic conductances of STN-to-GPe synapses and the GPe intrinsic noisy conductance. The GPe intrinsic noisy conductance (GPe Int. for short) was introduced to model autonomous activity of GPe neurons, and was implemented as a pseudo excitatory synaptic conductance that responds to a pseudo spike train obeying a Poisson process in order to reproduce both facilitating and desynchronizing effects on GPe neuronal activity. The mean rate of a pseudo spike train was set as 100 Hz, unless otherwise noted. The dopamine depletion-related reduction of GPe autonomous activity was modeled as the reduction of the maximal conductance of the GPe intrinsic noisy conductance. Although there were other ways to implement autonomous activity, such as using single-compartment or multi-compartment conductance-based models incorporating hyperpolarization-activated, cyclic nucleotide-gated (HCN) channels, we chose this simple implementation because our focus was generation of STN parkinsonian oscillations. The means by which significant variability in intrinsic electrophysiological properties of GPe neurons (Günay et al., 2008) affects generation of parkinsonian oscillations was beyond the scope of this paper.

### Short-term plasticity models

**Figure 2:**
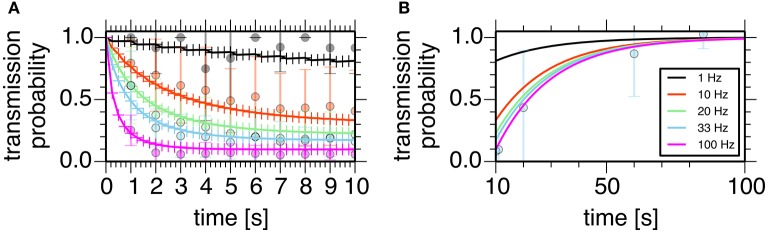
**Properties of the short-term depression model at a GPe-STN synapse**. Plots show simulated transmission probability of a single GPe axon stimulated at 1, 10, 20, 33, and 100 Hz for 10 s. **(A)** Depression during stimulation. **(B)** Recovery from the depression. Crosses represent simulation results, and circles with error bars represent means and standard deviations of experimental data (Atherton et al., 2013).

Short-term depression at a GPe-to-STN GABAergic synapse was simulated using a phenomenological model:

$$\tau_{D}\frac{dP}{dt}=1-P,$$

where *P* was the transmission probability of a unitary GPe projection. At every arrival of a presynaptic spike, *P* was decreased by multiplication with a decrement factor as follows:

$$P\to P\left[1+\left(f_{D}-1\right){\left(\frac{P-P_{bound}}{1-P_{bound}}\right)}^{n_{bound}}\right],$$

where the parameter *f*_*D*_ < 1 and the depression of *P* was limited by a lower bound, *P*_*bound*_. This implementation smoothly increased the decrement factor from *f*_*D*_ to 1 as the lower bound for *P* was reached, and smaller values of the parameter, *n*_*bound*_, retarded the increase of the decrement factor. At each synaptic contact of a unitary GPe projection, a spike was successfully transmitted with the probability,

$$\mu =1-{\left(1-P\right)}^{1/N_{contact}},$$

assuming that the transmission probability of a unitary GPe projection, *P*, obeyed the binomial distribution of transmissions at *N*_*contact*_ synaptic contacts made by a unitary GPe projection. Parameters of these models were manually tuned to fit experimental data (Atherton et al., 2013; Figure 2).

We used the phenomenological model by Hanson and Jaeger (2002) to simulate short-term plasticity at STN-to-GPe glutamatergic synapses. In this model, response amplitude, *A*, was a product of a facilitation variable, *F*, and a depression variable, *D* (*F* > 1 and *D* < 1) that decayed exponentially:

$$\begin{array}{l} \;A=F*D,\\ \tau_{F}\frac{dF}{dt}=1-F,\\ \tau_{D}\frac{dD}{dt}=1-D. \end{array}$$

At every arrival of a presynaptic spike, the facilitation variable, *F*, was increased through

multiplication by an incremental factor as follows:

$$F\to F\left[1+\left(f_{F}-1\right)\left(\frac{F_{bound}-F}{F_{bound}}\right)\right],$$

where the parameter *f*_*F*_ > 1 and the increase of *F* was limited by an upper bound, *F*_*bound*_. At every arrival of a presynaptic spike, the depression variable, *D*, was decreased by multiplication by the parameter, *f*_*D*_ (*f*_*D*_ < 1). According to Hanson and Jaeger (2002), each synapse was randomly assigned to one of three types of plasticity, facilitation-dominant (type 1, *f*_*F*_ = 1.4, *f*_*D*_ = 0.9, τ_*F*_ = 241.0 ms and τ_*D*_ = 491.0 ms), intermediate (type 2, *f*_*F*_ = 1.34, *f*_*D*_ = 0.86, τ_*F*_ = 345.0 ms and τ_*D*_ = 700.0 ms), and depression-dominant (type 3, *f*_*F*_ = 1.64, *f*_*D*_ = 0.55, τ_*F*_ = 148.0 ms, and τ_*D*_ = 764.0 ms) with the same probability regardless of a post-synaptic neuron.

### Data analysis and computer simulations

Oscillatory activity of neurons, and oscillatory correlations of pairs of neurons were estimated by the power and cross-spectral analyses of spike trains with the shuffling method (Rivlin-Etzion et al., 2006). The compensated power spectral density (PSD) was calculated by dividing the spectrum of the original spike train by the mean spectrum of locally (*T* = 175–225 ms) shuffled (*n* = 50) spike trains (Tachibana et al., 2011). A confidence level for compensated PSD was calculated based on the mean and standard deviation of the spectrum in the range of 270–300 Hz (Rivlin-Etzion et al., 2006). A cell was classified as “oscillatory” when its compensated PSD had values above the confidence level (*p* = 0.01) in at least two adjacent frequency bins of 1,000 Hz. The compensated cross-spectral density (CSD) was calculated by dividing the CSD of the original spike train by the mean CSD of globally shuffled (*n* = 20) spike trains. A confidence level for compensated CSD was constructed based on the range of 270–300 Hz. A pair of neurons was classified as having “correlated oscillations” if the compensated CSD contained at least two adjacent bins within a frequency range, the values of which were above the confidence level (*p* = 0.01). Bursts were detected by the “Poisson surprise” method (Wichmann and Soares, 2006). A Poisson surprise value of 3 for a given sequence of at least two interspike intervals (three spikes) was used to separate burst from non-burst spike trains.

All neuronal network simulations were performed in NEST (http://www.nest-initiative.org), and analyses of simulation results were performed in R and Python using Numpy, Scipy, Matplotlib, rpy2, and statsmodels libraries.

## Results

We constructed a spiking neuron model of the STN-GPe network by incorporating known physiological and anatomical properties of their neurons and synapses (Figures 1, 2, see Methods for details). Specifically, we incorporated short-term facilitation and depression of STN-to-GPe synapses (Figure 1B; Hanson and Jaeger, 2002) and short-term depression of GPe-to-STN synapses (Figures 1C, 2; Atherton et al., 2013). We considered changes in strengths of STN-GPe and GPe-STN synapses and intrinsic excitability of GPe neurons (GPe Int.) as parameters possibly affected by dopamine depletion, and thereby potentially responsible for the 8–15 Hz oscillatory bursts.

**Figure 3:**
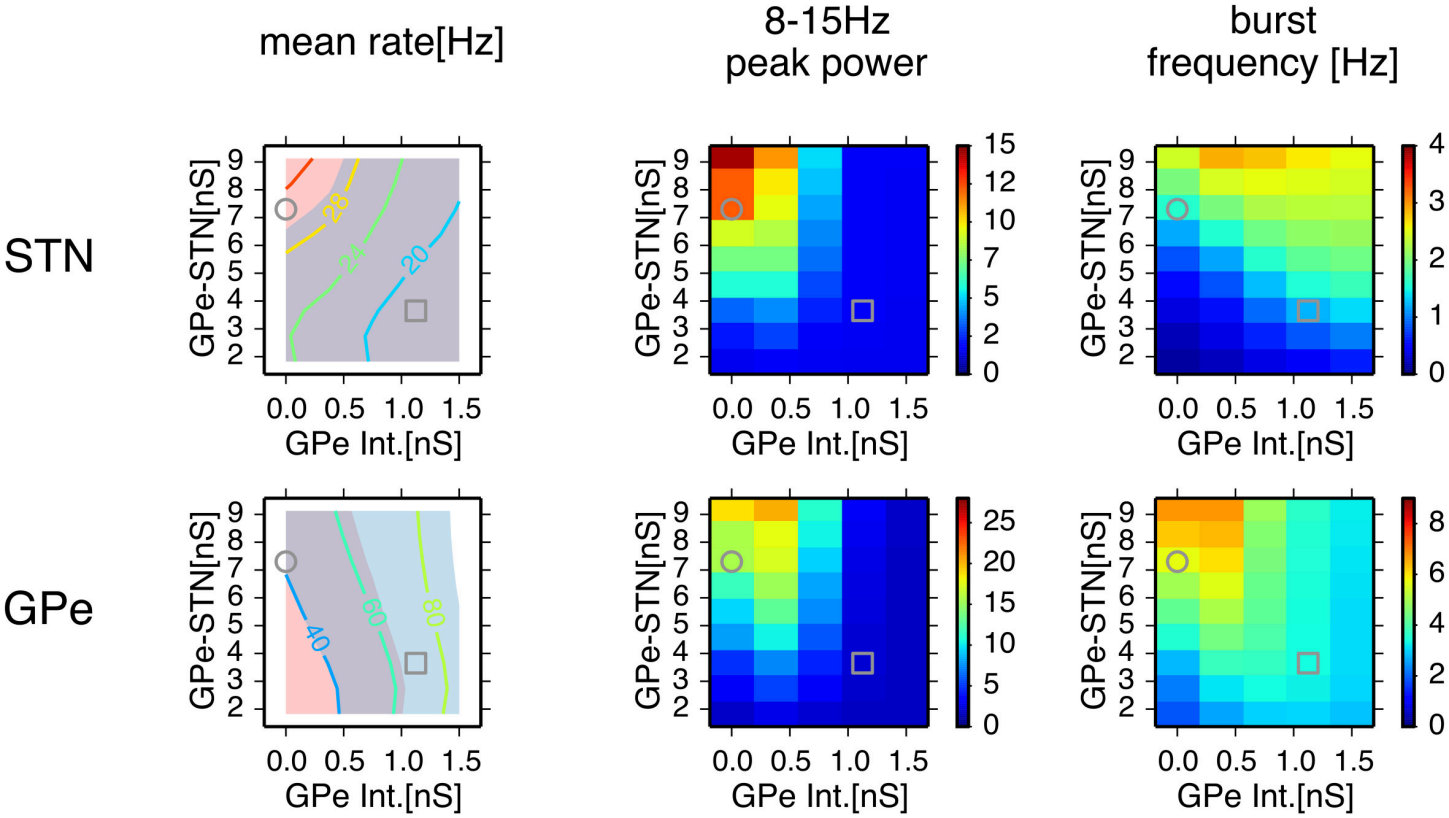
**Comparison of conditions for properties of neuronal activity with simulations of a network model for different parameter values**. A column of two panels corresponds to the feature of neuronal activity noted above. In each panel, each data point represents the mean of 8 distinct simulations with values of maximal conductances of GPe-STN synapses and GPe intrinsic noisy conductances (GPe Int.) that control levels of GPe autonomous activity, shown on the axes. In the left column, a contour plot in each panel represents the mean firing rates of neurons in simulation results, and light red and light blue patches behind the contour plot indicate parameter areas for which simulation results are within the means ± standard deviation of firing rates of neurons recorded from the BG of parkinsonian and normal monkeys (Tachibana et al., 2011), respectively. An overlap between light red and light blue patches appears slate gray. Grey circles and squares indicate parameters that correspond to the parkinsonian and normal states, respectively.

**Figure 4:**
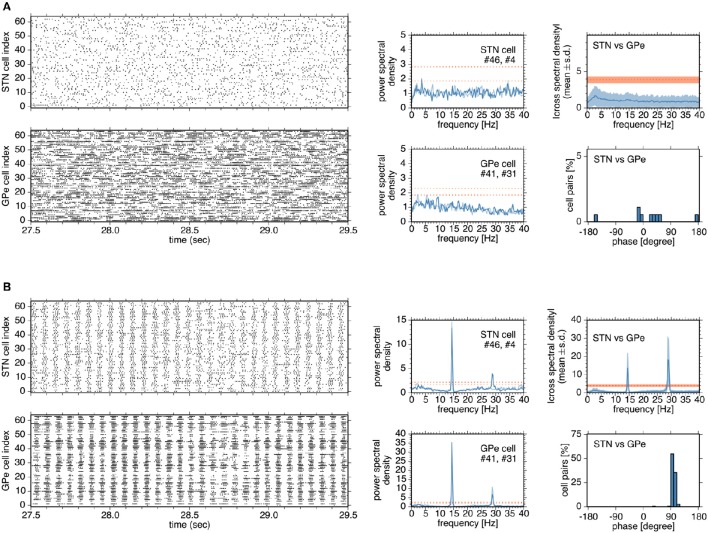
**Typical network and neuronal activities in simulations in the normal (A)** and parkinsonian states **(B) (A,B)**, Left, four panels show raster plots of STN and GPe neurons. Although a network model contains 192 neurons for the GPe, activities of 64 neurons are shown for the sake of clarity. In the middle column, each panel shows the power spectral density functions of two neurons in the STN or GPe. Heavy and light blue traces indicate the power spectral density functions of two neurons, respectively. Heavy and light red dotted lines indicate the confidence levels of the power spectral density functions of two neurons, respectively. In the right column, a top panel shows the absolute cross-spectral density functions of 196 randomly-selected pairs of STN and GPe neurons in a simulation, and a bottom panel shows the instantaneous phase values for the correlated pairs of STN and GPe neurons.

We systematically searched the parameter space for regions that reproduce neuronal behaviors reported in Tachibana et al. (2011), namely, the mean firing rate, the peak power of 8–15 Hz oscillation, and the number of bursts per second (burst frequency) in normal and dopamine-depleted monkeys (Table 1). We found that there are distinct parameter regions where simulated neuronal activities of STN and GPe exhibited properties similar to those experimentally observed in normal and parkinsonian states (Figure 3). In the normal state (ḡ_*AMPA*−*GPe*−*Int*_ = 1.125 nS, ḡ_*GABA*−*GPe*−*STN*_ = 3.65 nS, ḡ_*AMPA*−*STN*−*GPe*_ = 1.05 nS; open squares in Figure 3), electrophysiological properties of STN and GPe neurons of the model were similar to those in normal animals (Table 1), although the proportion of GPe neurons showing 8–15 Hz oscillation was >10x larger than those observed in animal experiments. The parkinsonian state was reproduced with a lower GPe intrinsic excitability and a higher GPe-STN synaptic strength (ḡ_*AMPA*−*GPe*−*Int*_ = 0.0 nS, ḡ_*GABA*−*GPe*−*STN*_ = 7.3 nS, ḡ_*AMPA*−*STN*−*GPe*_ = 1.35 nS; open circles in Figure 3), where STN and GPe neuronal activities showed higher burst frequency and stronger 8–15 Hz oscillations than in the normal state. In this parameter region, mean firing rates of simulated neurons in both structures (STN and GPe) were well matched to mean firing rates of neurons experimentally recorded from dopamine-depleted, parkinsonian-state monkeys (Tachibana et al., 2011, Table 1; Figure 3). The peak frequency of 8–15 Hz oscillations with maximum power ranged from 14 to 15 Hz in both structures (Figure 4B, Table 1). This range of peak frequencies is very similar to pathological oscillations observed in dopamine-depleted monkeys (Raz et al., 2000; Heimer et al., 2006; Rosin et al., 2011; Tachibana et al., 2011). No or very few neurons exhibited significant 3–8 Hz oscillations under the conditions tested.

**Table 1:** **Electrophysiological properties of STN and GPe neurons from animal experiments (A), and model simulations (B)**.

**STN**	**(A) Animal experiments**		**(B) Model simulations**	
	**Normal (*n* = 90)**	**Parkinson (*n* = 55)**	**Normal (*n* = 512)**	**Parkinson (*n* = 512)**
Firing rate (Hz)	19.9 ± 9.5	27.6 ± 11.3^*^	17.2 ± 1.70	31.6 ± 1.43^*^
Number of 8–15 Hz oscillatory cells (%)	5.5	36.3	0.0	100.0
Mean 8–15 Hz power	1.07 ± 0.12	1.19 ± 0.17^*^	1.01 ± 0.03	0.92 ± 0.05^*^
Peak power at 8–15 Hz	1.62 ± 0.27	2.01 ± 0.58^*^	1.54 ± 0.13	12.5 ± 2.3^*^
Mean frequency with peak power at 8–15 Hz (Hz)	11.4 ± 0.0	13.0 ± 1.4	NA	14.4 ± 0.1
Percentage of spikes in bursts (%)	54.1 ± 15.3	65.1 ± 10.6^*^	36.2 ± 11.2	33.4 ± 9.4^*^
Number of burst per second (Hz)	1.68 ± 0.61	2.73 ± 0.92^*^	1.31 ± 0.34	2.43 ± 0.97^*^
**GPe**	**Normal (*n* = 105)**	**Parkinson (*n* = 81)**	**Normal (*n* = 1,536)**	**Parkinson (*n* = 1,514)**
Firing rate (Hz)	65.1 ± 25.6	41.1 ± 22.3^*^	77.8 ± 17.1	56.6 ± 27.9^*^
Number of 8–15 Hz oscillatory cells (%)	0.9	6.1	16.0	95.4
Mean 8–15 Hz power	0.83 ± 0.12	0.97 ± 0.16^*^	1.06 ± 0.04	1.15 ± 0.23^*^
Peak power at 8–15 Hz	1.28 ± 0.22	1.51 ± 0.35^*^	1.68 ± 0.16	19.8 ± 10.0^*^
Mean frequency with peak power at 8–15 Hz (Hz)	NA	13.2 ± 0.6	11.7 ± 14.4	14.4 ± 0.1
Percentage of spikes in bursts (%)	37.6 ± 19.7	58.3 ± 19.2^*^	52.1 ± 9.6	68.5 ± 14.4^*^
Number of burst per second (Hz)	1.34 ± 0.74	2.06 ± 0.93^*^	3.25 ± 0.61	7.40 ± 2.83^*^

These values were recomputed from spike times recorded from normal and dopamine-depleted monkeys (Tachibana et al., 2011). Firing properties of STN and GPe neurons are represented as the mean ± SD. Mann-Whitney U-tests were performed between normal and parkinsonian (parkinson) states. Significance levels are as follows:

* *P < 0.001. NA, data not available. **(A)** Data from animal experiments with non-human primates of normal and dopamine-depleted, parkinsonian states (Tachibana et al., 2011). **(B)** Data from simulations of our model at normal and parkinsonian states shown in Figure 3*.

In the normal state, synchronous oscillatory burst discharges were not observed, and no neurons exhibited 8–15 Hz oscillations (Figure 4A). Burst discharges were also detected in the normal state, but they occurred in a rather irregular manner and less frequently, and their durations were longer than those in the parkinsonian state. In contrast, in the parkinsonian state, neurons showed synchronous oscillatory burst discharges, with different timing in different nuclei (Figure 4B). Each neuron exhibited 14–15 Hz burst oscillation and frequent burst firings. Cross-spectral density functions of pairs of STN and GPe neurons peaked at 14–15 Hz, as in the power spectral density functions of STN and GPe neurons (Figure 4B). Instantaneous phase differences for STN vs. GPe pairs clustered around 90°, indicating that the maximal firing of GPe neurons lagged behind that of STN neurons by ~17 ms (Figure 4B, right panels), which is comparable to experimental observations in rodents (Mallet et al., 2008).

### Mechanisms for generation of 8–15 Hz oscillatory burst firings

**Figure 5:**
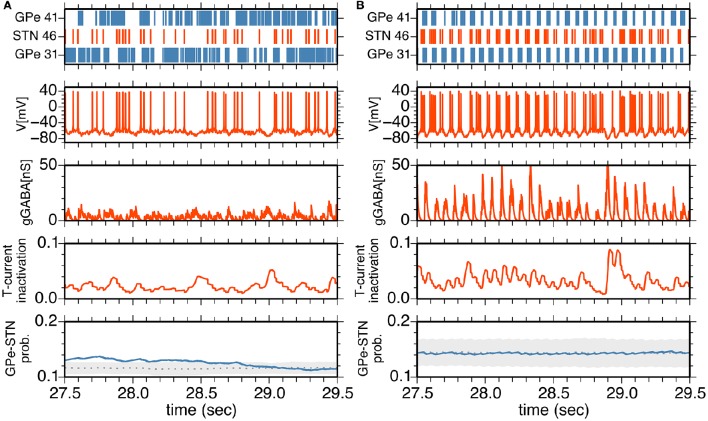
**(A,B)** Interactions between an STN neuron and its afferent and efferent GPe neurons in the normal **(A)** and parkinsonian states **(B)**. The top panel shows a raster plot of an STN neuron (red) and afferent and efferent GPe neurons (blue). The middle three panels show membrane potential, GABA-receptor-mediated synaptic conductance and an inactivation variable of T-current as a function of time for the STN neuron. The bottom panel shows the transmission probability of a unitary GPe projection to the STN neuron. A gray dotted trace, and light gray area indicate the mean and mean ± standard deviation of transmission probabilities of all GPe projections to the STN neuron. A blue trace indicates a transmission probability of the projection from the afferent GPe neuron as a function of time.

To understand neuronal and synaptic mechanisms underlying 14–15 Hz oscillatory burst firings in the parkinsonian state, we focused on a local sub-circuit in the STN-GPe network, an STN neuron (STN #46), an afferent (presynaptic) GPe neuron (GPe #41), and an efferent (postsynaptic) GPe neuron (GPe #31). Under normal conditions, STN, and GPe neurons did not exhibit clear synchronous oscillations (Figure 5A). GPe neurons showed sustained, high-frequency discharges with occasional pauses, and the STN neuron emitted isolated spikes in an irregular manner. In spite of high firing rates of presynaptic GPe neurons, their GABAergic influences on postsynaptic STN neurons were weak (Figure 5A) due to short-term depression of GPe-STN synapses (Figure 5A).

In the parkinsonian state (Figure 5B), STN neurons received strong inhibition due to burst discharges of their presynaptic GPe neurons, and were rapidly hyperpolarized so that T-currents of STN neurons were de-inactivated, as shown by an increase of the T-current inactivation variable (Figure 5B). This induced strong post-inhibitory rebound excitation of STN neurons. Consequent burst firing of STN neurons then strongly excited their postsynaptic GPe neurons to discharge in bursts. Timing of burst discharges of efferent GPe neurons overlapped with subsequent bursts of afferent GPe neurons (Figures 4B, 5B). Although an afferent GPe neuron seldom received a reciprocal excitatory connection from its postsynaptic STN neurons under the sparse random connection architecture, these synchronous bursts of GPe neurons shut off rebound burst firing of STN neurons and then hyperpolarized them again. By repeating this cycle of events, STN-GPe circuits maintained rhythmic burst discharges.

The timing of shut-off of STN burst discharges by inhibition from GPe neurons is critical for the 14–15 Hz oscillations because post-inhibitory rebound burst discharges could last longer without active shut-offs. The slow time scale of the post-inhibitory rebound mechanism alone could lead to much slower oscillations. Although almost none of the pairs of STN and GPe neurons in the network are reciprocally connected under the connection probabilities between STN and GPe neurons in the network model, network synchronization makes presynaptic and postsynaptic GPe neurons to an STN neuron discharge in bursts in a synchronous manner.

**Figure 6:**
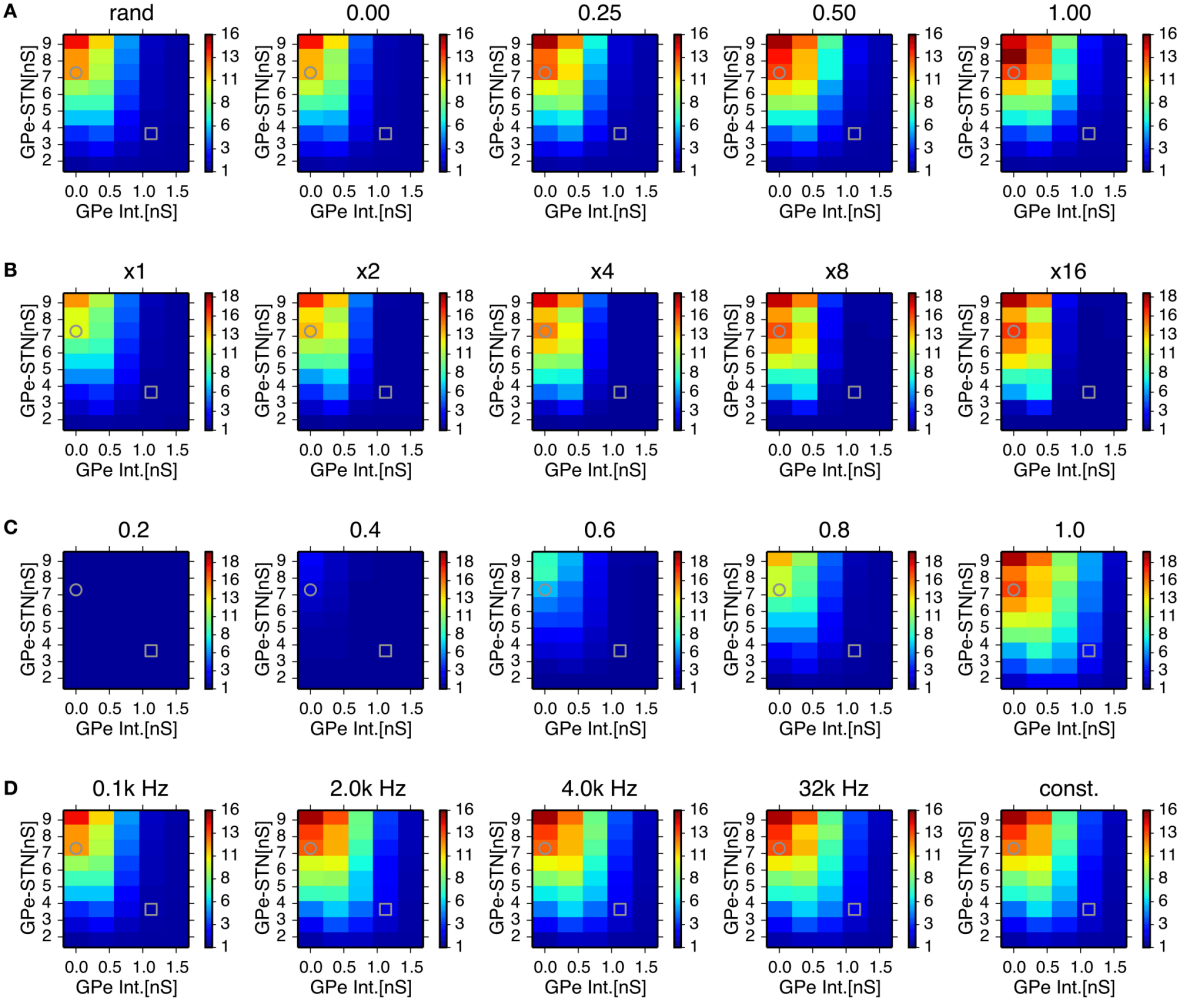
**Effects of probability of closed-loop connections between STN and GPe neurons (A)** sparseness in connections between STN and GPe neurons **(B)** properties of short-term depression at GABAergic synapses of STN neurons **(C)** and stochasticity in GPe intrinsic noisy conductances **(D)** on the 8–15 Hz oscillatory activity in the STN. Each panel represents dependencies of STN parkinsonian oscillations over maximal conductances of GPe-STN synapses and the GPe Int. in the same manner as in Figure 3 with a modified parameter value noted above. Grey circles and squares indicate parameters that correspond to the parkinsonian and normal states, respectively. **(A)** Probability of closed-loop connections between the STN and GPe neurons increased from near-zero probability of closed-loop connections as a result of the default connection rule, random-convergent connections, denoted as “rand.” **(B)** Sparseness in connections between the STN and GPe neurons was enhanced by scaling network size 2, 4, 8, and 16 times under the same connection rule. **(C)**, Strength of short-term depression at GABAergic synapses of STN neurons was enhanced by reducing values of the short-term depression parameter, *n*_*bound*_. **(D)**, Increasing the mean rate of a Poisson spike train noted above the panels with decreasing maximal conductance so as to keep the mean conductance constant over time, reduced the variation of GPe intrinsic noisy conductances. See details in the main text.

In order to assess the role of this sparsely closed circuit of STN and GPe neurons for the generation of oscillatory burst discharges, we ran simulations of network models with different proportions of reciprocally-connected pairs of STN and GPe neurons, while the connection probability was kept constant (Figure 6A). The power of 8–15 Hz oscillations increased with an increasing proportion of reciprocally connected neurons. However, oscillations remained even when reciprocal connections were absent.

We also tested whether synchronized bursts are due to highly convergent connections between STN and GPe neurons in the small network model we used. In simulations of network models with reduced connection probabilities between STN and GPe neurons by increasing the total numbers of STN and GPe neurons while maintaining the numbers of target neurons for each STN and GPe neuron constant, we observed stronger oscillatory burst activity (Figure 6B). This indicates that generation of oscillatory burst firings is not the result of the dense connectivity between STN and GPe due to the small network size.

STN-GPe network models with higher levels of GPe intrinsic excitability, including those in the normal state, exhibited much weaker oscillations in the 8–15 Hz range. Greater GPe intrinsic excitability can reduce GABAergic conductance of STN neurons due to the strong short-term depression of GPe-STN synapses (Figure 5B). The time constant of short-term depression was long enough to keep the synaptic transmission probability at STN GABAergic synapses almost fully depressed, indicating that short-term depression can act as a switch that turns on and off the 8–15 Hz burst oscillations in response to the level of presynaptic GPe activity. We tested the sensitivity of 8–15 Hz oscillatory burst discharges to the short-term depression parameter, *n*_*bound*_ (Figure 6C). Smaller *n*_*bound*_, which causes stronger depression, reduced the 8–15 Hz oscillations with lower levels of GPe intrinsic excitability, and larger *n*_*bound*_ enhanced the oscillations.

Another possible effect of higher GPe intrinsic excitability is enhanced stochasticity in GPe firing, because we implemented that using noisy conductance that obeyed a Poisson process (see Method). To address this issue, we investigated the effect of varying GPe intrinsic noisy conductance by increasing the mean rate of the Poisson process and reducing the maximal conductance inversely so as to keep the mean value constant. With decreased variance of GPe noisy intrinsic conductance, a slight enhancement of 8–15 Hz oscillations occurred at intermediate GPe intrinsic conductances (0.75 nS), but very little enhancement was observed at higher levels (1.5 nS), even when the conductance did not fluctuate stochastically, but remained constant at the mean value (Figure 6D). This result suggests that stochasticity in GPe intrinsic conductance is not a major factor in preventing the occurrence of 8–15 Hz oscillations.

### Roles of cortical excitatory inputs on STN parkinsonian oscillations

**Figure 7:**
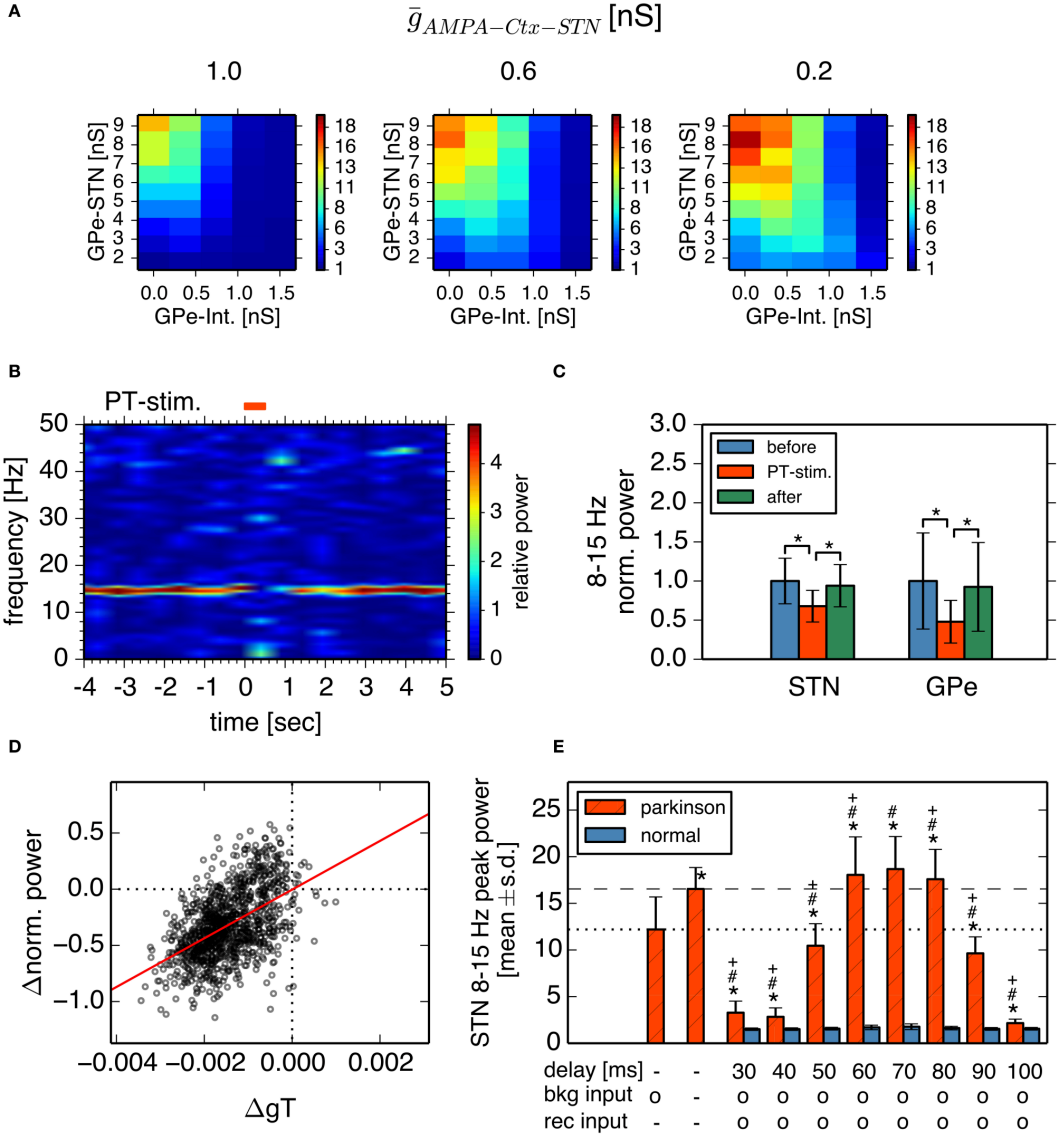
**Effects of cortical excitatory inputs on the STN 8-15 Hz oscillatory activity. (A)** Reduced background excitatory inputs to STN neurons in the model increase the peak power of STN 8–15 Hz neuronal oscillations. Each panel represents dependencies of the STN parkinsonian oscillations over the maximal conductances of GPe-STN synapses and GPe Int. in the same manner as in Figure 3. The maximal conductance of an AMPA-mediated synapse of an STN neuron that receives background excitatory inputs from the cortex, ḡ_*AMPA*−*Ctx*−*STN*_, noted above each panel. **(B,C)** Phasically increased excitatory inputs to STN neurons suppressed 8–15 Hz oscillations of STN and GPe neurons. **(B)** Typical spectrogram of an STN neuron in the model around 1,000 Hz excitatory Poisson spike inputs (PT-stimulation) mimicking peri-movement activations of cortical, layer-V pyramidal tract neurons. **(C)** Changes of mean power of 8–15 Hz oscillations of the STN and GPe neurons of the model by 1,000 Hz PT-stimulations. Blue, red, and green columns represent normalized power of 8–15 Hz oscillations of 1-s period before, just after, and 1-s after onset of PT-stimulation of 1,000 Hz, respectively. ^*^ indicates statistically significant changes (Friedman rank sum test, *p* < 0.001; pair-wise Wilcoxon rank sum test, *p* < 0.001, *n* = 256 neurons). **(D)** Correlation of changes of T-current conductances of and normalized power of oscillations of STN neurons by PT-stimulation. Each circle represents mean values of a neuron at a 250-ms bin during 1-s after onset of PT-stimulation. **(E)** Peak power of 8-15 Hz oscillations in the STN neurons depended on different GPi-thalamo-cortical delays of cortico-STN feedback oscillatory inputs. Each statistical value was calculated from pooled spike trains of STN neurons in 8 independent simulations (total *n* = 512 per condition). Red hatched and blue bars correspond to network parameters for the parkinsonian and normal conditions shown in Figure 3, respectively, except as otherwise noted. “delay” indicates the GPi-thalamus-cortex delays of AMPA-mediated recurrent feedback oscillatory inputs to the STN. Presence and absence of tonic background (bkg) and recurrent (rec) cortical excitatory inputs are shown as o and -, respectively. The leftmost bar represents data from the parkinsonian state without cortical recurrent inputs to the STN, shown in Figure 3, and the second bar from the left represents data of the parkinsonian state without either the tonic background or recurrent cortical inputs to the STN. Asterisks, hash marks, and plus marks represent significant differences (pair-wise *t*-test with Bonferroni adjustment, *p* < 0.001, *n* = 1,024) from the parkinsonian state without AMPA-mediated, recurrent oscillatory inputs to the STN, from the parkinsonian state without either the AMPA-mediated, background or recurrent oscillatory inputs to the STN, and from the parkinsonian state with AMPA-mediated, background inputs to the STN, and recurrent oscillatory inputs to the STN with a delay of 70 ms, respectively.

Next, we investigated how cortical excitatory inputs to the STN affect parkinsonian oscillations. Anatomically, layer-V pyramidal-tract neurons in the motor cortex are a major excitatory source for the STN (Parent and Parent, 2006; Kita and Kita, 2012). Layer-V pyramidal-tract neurons exhibit spontaneous firing and phasically increase their activities just before and during movements (Turner and DeLong, 2000; Pasquereau and Turner, 2011). First, we investigated the effects of cortical tonic background excitatory inputs to STN neurons on STN parkinsonian oscillations. We used homogeneous Poisson spike trains of fixed mean rate (100 Hz) as the background excitatory inputs to STN neurons, which were not correlated with BG parkinsonian oscillations. In contrast to experimental evidence (Tachibana et al., 2011), reducing synaptic strengths of background inputs to the STN enhanced 8–15 Hz neuronal oscillations in the STN (Figure 7A) and GPe (data not shown). This is because tonic excitatory inputs to STN neurons antagonized GPe inhibition, which depolarized STN neurons and diminished post-inhibitory rebound excitation. The suppressive effect of the background excitatory inputs on the parkinsonian oscillations suggests that stronger cortical excitatory inputs can reduce BG parkinsonian oscillations. This is consistent with experimental reports showing that pathological oscillations in LFPs recorded from the STN of PD patients were reduced by voluntary movements (Amirnovin et al., 2004; Brown and Williams, 2005) because just before and during voluntary movement, cortical inputs to the STN are phasically enhanced (Turner and DeLong, 2000).

Simulation results indeed showed that parkinsonian neuronal oscillations dropped at about the time of phasic increases (up to 1000 Hz) of excitatory inputs to STN neurons (Figures 7B,C). Reduction of STN parkinsonian neuronal oscillations correlated with reduction of STN T-current conductances that underlie post-inhibitory rebound excitation (*r*^2^ = 0.26, *p* < 0.001; Figure 7D). Smaller phasic increases (up to 250 Hz or 500 Hz) of excitatory inputs to STN neurons also showed drops of STN oscillations (Friedman test, *p* < 0.001; pairwise comparisons using Wilcoxon rank sum test, *p* < 0.001, data not shown).

We also investigated effects of oscillatory cortical inputs to STN neurons on STN parkinsonian oscillations. Because the manner in which cortical oscillations relate to STN parkinsonian oscillations is still unclear (Brittain and Brown, 2014), we simply assumed that STN neuronal oscillations propagate through the STN-GPi-thalamus-cortex loop and go back to the STN, while maintaining their oscillation frequency. We investigated the effect of the time delay in the STN-GPi-thalamus-cortex feedback loop on STN 8–15 Hz oscillations by introducing delay lines via which STN neurons were recurrently connected. More precisely, each STN neuron was randomly connected with 16 afferent STN neurons (average) and spikes were stochastically transmitted (*p* = 0.25) via delay lines. Synaptic strength of delayed feedback inputs was the same as that of background inputs to the STN. Simulation results showed that STN 8–15 Hz neuronal oscillations were affected by both the delay and the strength of the recurrent excitatory input to STN neurons (Welch's one-way ANOVA *F* = 4741.3, *df* = 17, *p* < 0.001). When the delay was within 60–80 ms, which was close to the cycle of STN parkinsonian oscillations, the STN oscillation in the parkinsonian state was stronger with feedback recurrent inputs than without (Figure 7E, pair-wise *t*-test with Bonferroni adjustment, *p* < 0.001, *n* = 512). In contrast, when the delay was shorter than 60 ms or longer than 80 ms, feedback recurrent inputs diminished STN oscillations to less than those without feedback. The power of STN oscillations in the parkinsonian state with feedback recurrent inputs, and with a delay between 60 and 80 ms was even greater than with a blockade of AMPA transmission both at background and at recurrent inputs to the STN (Figure 7E, pair-wise *t*-test with Bonferroni adjustment, *p* < 0.001, *n* = 512). Feedback excitatory inputs did not affect STN 8–15 Hz oscillations in the normal state (Figure 7E, pair-wise *t*-test with Bonferroni adjustment, *p* = 1.0, *n* = 512). These results indicate that cortical excitatory inputs affect STN parkinsonian oscillations in a complex manner: cortical non-oscillatory inputs are suppressive to STN parkinsonian oscillations, but feedback oscillatory inputs can amplify or diminish STN parkinsonian oscillations in a delay-dependent manner. The complex effects of cortical excitatory inputs on STN parkinsonian oscillations explain two lines of experimental evidence suggesting suppressive or facilitative effects of cortical-STN excitatory inputs, respectively (Amirnovin et al., 2004; Brown and Williams, 2005; Tachibana et al., 2011).

## Discussion

In this study, we explored mechanisms of generation of parkinsonian 8–15 Hz neuronal burst oscillations in the STN-GPe circuit by building a spiking neural network model, incorporating anatomical and physiological constraints. Through a systematic search of the parameter space, we identified parameter regions that reproduce normal and parkinsonian states as reported by Tachibana et al. (2011). The parkinsonian state was characterized by reduced intrinsic excitability of GPe neurons and increased conductance of GPe-STN synapses. Reduction of autonomous activity of GPe neurons by dopamine depletion paradoxically elevated inhibition of STN neurons via strong short-term depression and consequently induced strong post-inhibitory rebound burst firing of STN neurons, which were then shaped into synchronous oscillations in the 14–15 Hz frequency range via interactions between STN and GPe neurons.

Characteristics of neuronal activities observed in our simulations agreed well with experimental findings. First, generation of neuronal burst oscillations is critically dependent on GABAergic inhibition of STN neurons (Tachibana et al., 2011) and is sensitive to a level of GABAergic inhibition of STN neurons (Figure 3). Second, the frequency range of these neuronal burst oscillations is in the 8–15 Hz frequency band (Figure 4B), in which pathological oscillations were observed in the BG of dopamine-depleted monkeys (Heimer et al., 2006; Wichmann and Soares, 2006; Tachibana et al., 2011). Third, mean firing rates of neurons in the model exhibited in simulations under normal or dopamine-depleted states (Figure 3) accord well with those experimentally observed in the BG of monkeys (Tachibana et al., 2011). Fourth, inactivation of the STN diminishes 8–15 Hz neuronal oscillations in the GPe and GPi (Tachibana et al., 2011), which is consistent with our simulations with smaller synaptic strengths of STN projections (Figure 6). Fifth, acute administration of the dopamine precursor, L-DOPA, reduces the power of 8–15 Hz oscillations, the strength of burst discharges in the STN and GPi, and firing rates in the STN (Tachibana et al., 2011). These experimental results are consistent with our simulation results (Figures 3, 4) if L-DOPA reduces GABAergic inhibition of the STN by restoring D2-receptor-mediated presynaptic suppression (Shen and Johnson, 2000, 2005), but does not acutely elevate GPe autonomous activity through dopamine-dependent expression of HCN channels, which is supposed to be a slower process (Chan et al., 2011). Sixth, burst frequency in the STN and GPe (but not in the GPi) in the model were higher in the pathological state than that in the normal state (Figures 3, 4), which is consistent with experiments (Wichmann and Soares, 2006; Tachibana et al., 2011). Seventh, our simulation results suggest that contributions of cortical excitatory inputs to the STN for PD burst oscillations are complex and depend on their temporal patterns and firing rates (Figure 7). Enhancing non-oscillatory cortical inputs suppressed STN PD oscillations, which is consistent with experimental evidence from PD patients (Amirnovin et al., 2004; Brown and Williams, 2005), although there are also experimental results showing that the blockade of ionotropic glutamatergic transmission in the STN diminished STN PD oscillations (Tachibana et al., 2011). Additional oscillatory inputs, the frequency of which was similar to STN PD oscillations, could amplify or diminish STN PD oscillations in a phase-dependent manner. These amplifying effects of oscillatory cortical inputs are consistent with experimental results (Tachibana et al., 2011).

We verified the critical role of strong post-inhibitory rebound excitation of an STN neuron for generation and maintenance of PD 8–15 Hz neuronal burst oscillations (Figure 5). The same mechanism was already explored in the pioneering study of a spiking neuron model for PD oscillations (Terman et al., 2002), which explained the slower oscillations, but not the 8–15 Hz oscillations. The slowly-changing nature of the rebound mechanism is ascribed to the slower oscillations (Holgado et al., 2010). In our model, in response to STN rebound firing, GPe neurons actively terminate the rebound excitation of the STN despite sparsely closed loops between STN and GPe neurons, where each population was synchronously activated. This network mechanism enabled faster neuronal oscillations in spite of the slow rebound mechanism. The suppressive role of excitatory inputs on the STN for the 8–15 Hz oscillations we observed can be explained as the damping of the post-inhibitory rebound excitation by preventing required hyperpolarization. It is consistent with peri-movement, phasic activation of layer-V pyramidal tract neurons in the motor cortex projecting to both the spinal cord and the STN (Turner and DeLong, 2000). It is also consistent with the therapeutic effects of deep brain stimulation to the STN that is able to suppress 8–15 Hz oscillations by virtue of its high-frequency, direct stimulation of cortical afferents to the STN (Miocinovic et al., 2006).

The decline of GPe autonomous activity due to down regulation of the HCN channel caused by dopamine depletion (Chan et al., 2011) is the key phenomenon in our model for converting normal neuronal activity into a pathological state. Normal autonomous activity of GPe neurons could have two possible effects: maintenance of a high level of neuronal firing, and decorrelation of GPe neuronal activities by collateral inhibition (Edgerton and Jaeger, 2011). Our simulations showed that GPe neuronal firing maintained at a high level reduces GABAergic inhibition of the STN via strong short-term depression (Atherton et al., 2013) and prevents the generation of pathological oscillations. Once GPe neuronal firing is reduced due to reduced autonomous activity caused by dopamine depletion, GABAergic inhibition becomes less depressed and induces the rebound excitation of STN neurons, and consequently pathological oscillations occur. Decorrelated GPe neuron activities may prevent the generation or maintenance of 8–15 Hz burst oscillations, but it showed limited effects in a PD state in our simulations. Decorrelation of GPe neurons might be more implicated in normal BG functioning.

Although elevated striatal inhibition of GPe neurons by dopamine depletion (Liang et al., 2008) has been considered the key cause of PD pathologies (Albin et al., 1989; DeLong and Wichmann, 2007), we did not explore its role relative to pathological oscillations because of negative experimental evidence showing that blockade of ionotropic GABAergic transmission in the GPe does not change or elevate the power of the pathological oscillations (Tachibana et al., 2011). However, elevated striatal inhibition could have a similar effect, since the decline of GPe autonomous activity in our model reduced GPe firing to cause pathological oscillations.

Our simulation results showed that in-phase oscillatory excitatory inputs from the cortex amplify pathological oscillations in the STN, as observed experimentally (Tachibana et al., 2011). Indeed, cortical and STN oscillatory activities are mainly coherent in the low (12–20 Hz) and high (20–35 Hz) beta range (Hirschmann et al., 2011). Whether STN PD lower-beta oscillations proliferate throughout the cortex-BG-thalamus loops as an entrainment loop is currently a matter of debate (Brittain and Brown, 2014). However, enhancement of cortical lower-beta oscillations in PD patients (Pollok et al., 2012) and neuronal lower-beta (and upper-beta) oscillations of pyramidal tract neurons in the motor cortex of dopamine-depleted primates (Pasquereau and Turner, 2011) are consistent with the entrainment loop hypothesis. The required delay from the STN to the cortex might be too large considering just conduction and synaptic delays. However, because GPi projections to the thalamus are inhibitory, oscillations could be delayed by more than the sum of conduction and synaptic delays, as observed in closed STN-GPe circuits. The reduction of STN PD lower-beta oscillations by out-of-phase cortical oscillations in our simulations is a result of damping of STN post-inhibitory rebound excitation by counteraction of phasically-elevated excitatory inputs from the motor cortex to inhibition from the GPe. It is similar to the suppression by dynamically increased cortical inputs during movements (Turner and DeLong, 2000), and account for mechanisms of closed-loop deep brain stimulation (Rosin et al., 2011).

Our model has several limitations in reproducing experimentally observed phenomena. First, our model did not show neuronal oscillations in the 3–8 Hz frequency band in the BG of dopamine-depleted monkeys (Heimer et al., 2006; Wichmann and Soares, 2006; Tachibana et al., 2011), although the relationship of such oscillations to dopamine depletion is controversial. Second, burst frequency of the STN in the parkinsonian state in our simulations was lower than in experimental observations (Wichmann and Soares, 2006; Tachibana et al., 2011).

In summary, our model provides plausible mechanisms for generation and maintenance of PD oscillations in the BG. Our model also showed that cortical inputs to the STN can amplify PD oscillations and it predicted required conditions for amplification or reduction of pathological BG oscillations.

## Author contributions

OS and KD planned the study. YT and AN provided physiological data. OS constructed the model and performed numerical simulation. All authors discussed the results and OS and KD wrote the manuscript.

### Conflict of interest statement

The authors declare that the research was conducted in the absence of any commercial or financial relationships that could be construed as a potential conflict of interest.
